# Epistemic racism in the health professions: A qualitative study with Black women in Canada

**DOI:** 10.1177/13634593221141605

**Published:** 2022-12-07

**Authors:** Brenda L Beagan, Stephanie R Bizzeth, Kaitlin R Sibbald, Josephine B Etowa

**Affiliations:** Dalhousie University, Canada; Dartmouth General Hospital – Nova Scotia Health, Canada; Dalhousie University, Canada; University of Ottawa, Canada

**Keywords:** ethnicity and health, profession and professionalization, race, social inequalities in health

## Abstract

Systemic racism within health care is increasingly garnering critical attention, but to date attention to the racism experienced by health professionals themselves has been scant. In Canada, anti-Black racism may be embodied in structures, policies, institutional practices and interpersonal interactions. Epistemic racism is an aspect of systemic racism wherein the knowledge claims, ways of knowing and ‘knowers’ themselves are constructed as invalid, or less credible. This critical interpretive qualitative study examined the experiences of epistemic racism among 13 healthcare professionals across Canada who self-identified as Black women. It explores the ways knowledge claims and expert authority are discredited and undermined, despite the attainment of professional credentials. Three themes were identified: 1. Not being perceived or portrayed as credible health professionals; 2. Requiring invisible labour to counter professional credibility ‘deficit’; and 3. Devaluing knowledge while imposing stereotypes. The Black women in our study faced routine epistemic racism. They were not afforded the position of legitimate knower, expert, authority, despite their professional credentials as physicians, nurses and occupational therapists. Their embodied cultural and community knowledges were disregarded in favour of stereotyped assumptions. Adopting the professional comportment of ‘Whiteness’ was one way these health care providers strived to be perceived as credible professionals. Their experiences are characteristic of ‘misogynoir’, a particular form of racism directed at Black women. Anti-Black epistemic racism constitutes one way Whiteness is perpetuated in health professions institutions.

## Introduction

The impact of racism on individual and population health in Canada has been well-documented (Dryden and Nnorom, 2021), but far less attention has been paid to experiences of racism within the health professions themselves. This has begun to change, with recent evidence emerging about systemic racism in occupational therapy, physiotherapy and medicine (e.g. Beagan et al., 2022a, 2022b; Dhara, 2020; Hughes et al., 2021; Mocanu et al., 2020; Mpalirwa et al., 2020; Vazir et al., 2019). This adds to the many years of evidence of systemic racism in the Canadian nursing profession (Bouabdillah et al., 2021; Calliste, 1996; Das Gupta, 1996; Etowa et al., 2009; Jefferies et al., 2018; Modibo, 2004; Onagbeboma, 2020; Premji and Etowa, 2014; Saleh, 2017; Vukic et al., 2012). When racism is systemic – foundational to and pervasive throughout a society – it manifests across social contexts at multiple levels, including interpersonal, institutional and structural (Jones, 2000; Nazroo et al., 2019). Thus it is not unexpected to see systemic racism documented within the health professions.

One of the forms racism may take is epistemological. Epistemology refers to knowledge systems and rules regarding what can be known and how, what ways of knowing are considered valid and who can be a legitimate and authoritative knower (Zaidi et al., 2021). These knowledge-rules are often implicit, and often tacitly racialized. In Canada, the voices and knowledge claims of White Western scholars, researchers and professionals have long been privileged. It has been argued that the epistemologies governing health professions in Canada are built on the knowledges of a handful of White men from five Western European countries (Grosfoguel, 2013; Paton et al., 2020). This is solid grounding for pervasive epistemic racism, wherein the knowledge held and shared by people of colour is automatically discredited (Paton et al., 2020). White-Euro-Western ways of knowing and forms of knowledge are legitimated and granted authority, while all others are rendered inferior. Epistemic racism can also mean particular knowers *themselves* are devalued. When the knowledge claims upon which professional authority is granted do not extend to certain kinds (or colours) of knowers, certain epistemic agents, due to identity-prejudice, a ‘credibility deficit’ occurs (Dotson, 2011). Those knowers are deemed to inherently lack credibility.

Anti-Black racism is a distinct form of racism, grounded in histories of colonization and slavery and forming a contemporary cornerstone of racialized capitalism (Dryden and Nnorom, 2021; Smith, 2010; Tyler, 2020). Within healthcare, Black professionals face racism at all levels. In a recent Canadian survey of Black physicians and residents, over 70% reported negative experiences due to racism (Mpalirwa et al., 2020). They were routinely taken for floor aides or housekeeping staff, patients refused to receive care from them and ‘their competence was occasionally called into question with patients not acceding with their plan until a White physician agreed with it’ (Mpalirwa et al., 2020, p. S53). This is epistemic racism, where credibility and legitimacy are undermined by identity-based prejudice.

Anti-Black epistemic racism also occurs when Black people are ignored, required to change how and what they say, not consulted, or excluded from decision-making positions (Collins, 2000; Dotson, 2011, 2012; Mills, 2007). While the Whiteness that dominates health professions education, curricula and research is troubling (Grenier, 2020; Hughes et al, 2021; Paton et al., 2020), it is compounded by the absence of Black people in positions of authority within their professions (Bouabdillah et al., 2021; Jefferies et al., 2018; Modibo, 2004; Premji and Etowa, 2014). That absence both relies on and perpetuates perceptions that Black voices – Black people – lack credibility, lack expertise, and lack legitimate professional authority.

While anti-Black racism is clearly experienced across genders, its intersection with gender hierarchies means women experience it differently (Bailey, 2021; Collins, 2000). Bailey (2016, 2021) calls this distinct intersection ‘misogynoir’. Stereotypes casting Black women as overly emotional, angry, strong, impervious to pain, sexualized, indefatigable workers and domineering matriarchs shape how Black women are perceived (Collins, 2000; Etowa et al., 2017); these stereotypes are reproduced in health care discourses that establish White bodies, knowledges and emotions as normative, producing Black women as Other (Bailey, 2016). Though epistemic racism can be clearly located in institutional processes, such as pedagogy and curricula, it is also important to attend to its ‘agential components,’ the ways it operates through everyday interpersonal interactions (Medina, 2017: 42). This paper examines how Black women healthcare providers encounter anti-Black epistemic racism within their roles as health professionals.

## Methods

Our critical interpretive qualitative study examined the experiences of 13 healthcare providers who self-identified as Black women: 9 nurses, 3 occupational therapists and 1 physician. After research ethics approval was obtained from three universities, participants were recruited from across Canada, using snowball sampling, recruitment posters and social media platforms. Participants were required to have at least 5 years practice experience in Canada. Those who responded to recruitment information were emailed the study details and consent forms, and their eligibility was confirmed. We report only high-level demographics (Table 1) to preserve confidentiality.

**Table 1.** Demographics.

Characteristic	Category	*N* = 13
Age	30s	3
	40s	6
	50s	4
Years in practice	5–9	2
	10–14	2
	15–19	4
	20+	5
Location	Rural/town	2
	Small/mid-sized city	7
	Large city	4
Practice setting	Hospital	9
	Private practice	2
	Community	2
Racialized identity	Black Canadian	5
	African or Caribbean migrant	8

After discussion of consent, individual, semi-structured interviews were conducted by phone or in person, by one of three interviewers. Each interview took 60–90 minutes, exploring experiences of belonging and marginality, as well as coping and resistance. Though we followed the lead of each participant, we used an interview guide that included questions about deciding to enter the profession, experiences during training, when they most and least felt they belonged in professional contexts, participants’ own expectations and the expectations others had of them and coping strategies when they encountered marginalization. Such topics can be emotional, and ethically we committed to supporting participants who became distressed. Unfortunately, the racism faced by Black health professionals was not a new experience or story for participants; strong emotions arose, including anger, but nothing beyond what they experienced everyday at work.

Interviews were audio-recorded, transcribed verbatim, then checked for accuracy while all identifying information was removed. Thematic analysis began with reading and re-reading transcripts, then moved to coding using ATLAS.ti qualitative data analysis software to facilitate team processes and transcript management. Some codes drew from theory and literature, while others were identified through repeatedly reading the data. Iterative analysis moved between compiling coded data and re-reading full transcripts, moving between theory and data and comparing experiences and interpretations across narratives. Quotations were organized and reorganized as sub-themes emerged, then ‘cleaned’ by removing false starts and filler words like ‘um’ and ‘ah’ for readability.

While we began by attending to racism, the specific framework of epistemic racism gradually emerged through ongoing team analysis and discussions, providing an overarching thematic structure for this paper. Weekly team meetings over many months focussed on systematic data interpretation, focussed on how we were conceptualizing our codes, whether we needed new codes to capture the data and how codes might need to be sub-divided for greater nuance, or merged to acknowledge a common idea (Braun and Clarke, 2021). Gradually our weekly discussions engaged more with theory and data interpretation.

Our regular meetings enabled a form of ‘transpersonal reflexivity’ (Dörfler and Stierand, 2020), team members ‘thinking together’ (p. 788) so that perceptions, experiences and beliefs become sources of interpretive insight. Lived experience informed every aspect of the research as we strived to mobilize our ‘biases’ and perspectives to enrich the analyses. The research team included Black, White and Indigenous members, and all team members have first-hand experience of some forms of marginalization in their professions. The team also included researchers from nursing, medicine and occupational therapy.

Conducting only single interviews with participants on this complex topic is the major limitation of this study. While it was intended to minimize participant burden for busy clinicians, it remains likely that some participants could have gone deeper in their reflections with a second interview. Secondly, while the inclusion of multiple professions in the sample enabled a focus on the processes of epistemic racism that cross specific contexts, at the same time it meant very small numbers from each field, which may mean we missed profession-specific details. While thematic saturation is often seen as a gold-standard for rigour in qualitative research (Guest et al., 2006), we would argue that data saturation is not really possible in interpretive research, it is a post-positivist holdover (Braun and Clarke, 2021). Data analysis spirals ever deeper, uncovering layers of meaning and interpretation. Nonetheless, the notion of ‘information redundancy’ – beginning to hear common narratives in subsequent interviews – is familiar to anyone who has conducted qualitative data collection, and we certainly had that experience. Thus, although we would argue the small sample size coupled with sample heterogeneity by profession) is a potential limitation, we also note the strength arising from a sample focussed solely on Black women health professionals, who were able to provide rich descriptions of the pervasive, endemic epistemic racism intersecting with gender power relations across multiple professions. Finally, as noted above, the biases brought to the study by our team – biases of positionality in race relations, and biases of professional affiliations – also constitute a strength, allowing insider as well as outsider perspectives in our analyses.

## Results

Below we examine three main themes: 1. Not being perceived or portrayed as credible health professionals; 2. Requiring invisible labour to counter professional credibility ‘deficit’ and 3. Devaluing knowledge while imposing stereotypes.

### Not being perceived or portrayed as credible health professionals

Participants described how they were not perceived as credible health professionals in medical spaces. Some ways this occurred was being mistaken for students or cleaning staff, having patients refuse to work with them and being overlooked or ignored. They were questioned and challenged more than their White colleagues, and rarely if ever saw Black people in management positions.

Numerous participants described being mistaken as cleaning or food service staff. Others were frequently mistaken for students, even when they had years of practice experience: ‘I often get mistaken for a student. . . I had a nurse come up to me and say “Hey, I’ve got this great opportunity for you to practice blood pressure”’. The participant saw this as ‘not being taken seriously’, and ‘I think that has more to do with my skin colour than how young or old I look’. One nurse said she was frequently asked, ‘When did you graduate? Are you qualified?’

Several participants described being scrutinized constantly by managers and instructors, their knowledge and expertise seemingly always in doubt. Almost all participants had experiences with patients refusing to work with them. Some participants had been deliberately targeted for scrutiny, with even the most minor oversights documented, to undermine their competence: ‘That period there was a lot of Black nurses that were being fired. And they were firing them over little. . . minor things that they would never have fired anybody else over’.

Some participants found their ideas were both appropriated and ignored, noting, ‘People come to me, one-on-one, pick my brain, and get ideas and I notice that sometimes they take those ideas as their own without acknowledging where they are from’. As one occupational therapist stated, ‘I guess one of the consequences of being racialized, and also a woman, is that I feel like my opinion isn’t taken into consideration’.

Those who spoke with other-than-Euro-Canadian accents reported being ignored, bullied, seen as less competent, their speech publicly ‘corrected,’ and/or overtly dismissed as incomprehensible by colleagues. One woman had her boss correct her accent while she was presenting to a room full of coworkers: ‘He took it upon himself to correct my language, in front of everybody. I tried to tell him that I don’t want to engage in that right then, but he didn’t [stop]. I felt that it was some kind of bullying’. She noted that White staff members with near-incomprehensible regional accents were never ‘corrected’.

One participant described imagery in the hospital that she thought showed professional authority and expertise are constructed as incommensurable with Blackness:

In the hallway, they have pictures of some people who are doing stuff, like the doctors, the technicians and all that. They put all the White people doing good things, and they put a Black male with a vacuum, or mop. Yeah. (laugh) Yeah. . . . We brought it to HR. But they never did anything. It is still there.

Professional authority was also undermined when managers never assigned Black nurses as charge nurse, giving this role to less-experienced White nurses: ‘She would not make any one of us charge nurse. It was bothersome, to see a new nurse who has no clue being in charge, and they come to ask you for advice’. One Black woman who *had* been charge nurse found White colleagues directly resisted her authority: ‘I had coworkers that refused to take my direction. One shift, I was in charge and I assigned a patient to a particular individual, who came to me to say, “I don’t take those patients. . . I don’t take direction from you’”. The idea that professional authority cannot be embodied by a Black woman was entrenched by seeing virtually no Black women in management positions.

Elsewhere in the hospital, there’s between ¼ and 1/3 of the staff that is visible minority. But not in management. There is no person in leadership or management or a VP role [who is] a visible minority. None. Not even nursing facilitators. . . Not one manager.

### Requiring invisible labour to counter professional credibility ‘deficit’

Starting from a dominant presumption that Black women cannot legitimately embody professional authority, as knowers and experts, our participants had to engage in ongoing, invisible labour to garner respect, to counter the ‘credibility deficit’ that attaches to devalued identities. They were required to change their appearances, shape their communication styles and acquire more education to be taken seriously.

To boost credibility, some employed dominant assumptions about professional comportment and appearance, such as always dressing up, or straightening their hair: ‘Some people would interpret my curly big hair as unprofessional’. Several participants described being told they were ‘too much’ for professional culture: Too loud, too angry, too emotional, too intense. One noted that even when raising issues of racism, she has to do it the ‘right’ way to be heard: ‘[I’ve been told] if you’re going to talk about racism, it depends how you bring it up. If you bring it up all angry and things, then it’s normal that no one would want to listen’. She went on, ‘There’s a lot of rules. . . I have to be careful how I talk about it. Everybody needs to still be comfortable around the topic and if they’re not comfortable, then I have to drop it (laugh)’. This was echoed by a participant who said, ‘I’ve got to bend myself in a pretzel, to make sure that they don’t feel uncomfortable’. Others learned to couch their contributions in ways that gave them added authority. For example, one occupational therapist mobilized evidence-based practice to gain credibility for her contributions: ‘I’ll talk about the evidence. . . always try to say things in a way that will be perceived by others as valid’.

Virtually all participants argued that as Black women they need to work twice as hard to prove to others they are equal to White colleagues: ‘In my work life probably I work harder than others. . . if I was just working as hard as everybody else probably I wouldn’t have the position I am in’. Participants described always being extra-prepared; one suggested that Black nurses need years more experience or advanced degrees before others will accept their competence.

You’ve gone for your Master’s and so forth, so then that’s paper that kind of shows, ‘Okay, I might not be working twice as hard, but this paper says I know twice as much’. . . The importance of that paper, (laugh) especially being Black, because you have to show that you’re more educated as well, in order for you to be taken a bit more seriously.

### Devaluing particular knowledge while imposing stereotypes

Some participants described how, although they brought knowledge of some of the specific challenges patients face, this knowledge was dismissed, while Black stereotypes were imposed.

For example, one nurse explained how culture shock, fear and isolation could mimic psychosis in patients from her cultural group, but ‘when you try to explain to [the team], they don’t consider your input’. Another participant argued that none of the assessments or interventions available to her took the health impacts of racism into account: ‘Like, depression is a thing, but let’s not talk about how racism might be contributing to that in any way!’ A physician found there was no space to act on community-based knowledge because of health system expectations. She described meeting with a Black family, low income, isolated and facing racism and knowing their complex constellation of needs ‘didn’t fit into the 15-minute time slot that everyone else in the clinic did. . . they needed more time’. She felt she was perpetuating systemic racism, poorly serving Black communities.

The expertise of Black women seemed to be denied even about racism. One occupational therapist had been challenged by a psychologist who disagreed with her assessment that some diagnoses among Black youth were as much a product of systemic racism as neurodiversity. The psychologist threatened a formal complaint: ‘She didn’t see someone with many years’ experience and a graduate degree and all this knowledge. . . Instead, she saw an ignorant Black woman and wanted to shut me down’.

At the same time, stereotypes were employed regarding the knowledges Black health professionals were expected to embody, such as speaking the same language as racialized patients, withstanding more violence and working well with all cultural minorities. For example, one nurse who wore hijab was routinely assumed to be able to speak with particular patients: ‘Whenever there is a patient wearing hijab, they will come and ask me if I speak their language. . . . Most of the time, I don’t. . . There is always assumptions. . . just because we look alike’. Laughing, she pointed out, ‘They assume that they [the patients] don’t speak English. And I’m like, ‘Well, did you even *try* to talk with them?’ (laugh) Maybe they speak fine English!’

In addition, a participant reported that all ‘cultural minority’ patients, especially non-English speakers, were assigned to racialized nurses, assuming they could connect culturally even without shared language. Some participants reported that violent patients tended to be assigned to Black providers. One nurse reported that Black patients were assigned to her because White nurses were ‘uncomfortable’ with them, ‘especially if it was a gunshot wound or any kind of gang relation, they always assigned the Black patients to me’.

## Discussion

The Black women in our study faced routine epistemic racism, not afforded the position of legitimate knower, expert, authority, despite their professional credentials as physicians, nurses and occupational therapists. They were questioned and undermined, their contributions ignored and/or appropriated. They did not appear to be perceived as ‘management material’ and those who held any management roles found their authority directly flouted. Our participants had to engage in invisible work and/or seek extra credentials in order to earn the perception of competence, a perception that appeared to be granted automatically to White colleagues. In turn, participants engaged in impression management, striving to embody White notions of professional comportment. Meanwhile the augmented knowledges they brought to their professions – their ‘extra,’ embodied cultural and community knowledges – were devalued and dismissed, displaced by stereotyped assumptions about what knowledges they *should* possess. These are instances of epistemic racism, wherein Black women may not be perceived as legitimate knowers, authorities, even when holding professional credentials and even when addressing realities with which they have direct first-hand experience (Dotson, 2012).

It may be tempting to see the experiences described here as being about ‘cultural differences’ rather than racism. Yet note that participants themselves refuted this analysis. They reported that Black women were dismissed for their accents, while White cultural-minority colleagues with equally challenging accents were not. Participants dismissed as being too loud, too intense and so on, were not solely immigrant women from other cultures, but also women with roots in Canada stretching back generations. Culture intersected racism at times – such as assigning cultural minority patients to Black healthcare providers, or assigning patients wearing hijab to professionals wearing hijab, assuming they would speak the same language – but the link is less about culture than about seeing both patient and provider as Other. That Othering is a component of epistemic racism.

In the context of epistemic racism, the knowledges of marginalized groups get little uptake within existing power structures. They are dismissed, rendered irrelevant. Writing about epistemic racism, Mills (2007) argues that failures to recognize and respect the personhood of another are part of the relations of dominance and subordination. Epistemic racism establishes legitimate and illegitimate knowledges, as well as two distinct classes of epistemic agents: knowers and sub-knowers (Pohlhaus, 2017: 17). This distinction is maintained in part through devaluing the credibility of certain knowers (Dotson, 2014). As Pohlhaus (2017) argues, ‘In such cases, an epistemic agent is unfairly prevented from participating fully within epistemic systems owing to an unfair distribution of epistemic power due to unwarranted credibility deficits and assessments of competency’ (p. 20). Our participants’ accounts definitely indicated such unwarranted credibility deficits.

Our participants’ experiences also exemplify misogynoir, ‘the co-constitutive, anti-Black, and misogynistic racism directed at Black women’ (Bailey, 2016: 2) which infuses not only popular social discourses but also medical and health professional discourses. As Bailey (2016) argues, change requires ‘looking closely at the ways that bias and prejudice are institutionalized in institutions of professionalization’ (p. 21). She urges epistemic multiplicity, making space for multiple ways of knowing, with multiple knowers granted credibility. Black health professionals are subject to heightened scrutiny, doubt, suspicion; in White-centric institutions, there is a predisposition to assume incompetence (Beagan et al., 2022a, 2022b; Bouabdillah et al., 2021; Mocanu et al., 2020; Mpalirwa et al., 2020; Vazir et al., 2019). At the intersection of anti-Black racism and misogyny, this appears especially true for Black women (Bailey, 2016; Collins, 2000; Etowa et al., 2009; Mocanu et al., 2020).

The narratives in our data seem remarkably persistent, echoing eerily the interview data collected by the fourth author over 20 years ago (Etowa et al., 2009). Though it is difficult to imagine greater numbers of Black health professionals is a solution, given the racism visited upon them at multiple levels, there is benefit to developing a critical mass, which may begin to undermine the Whiteness of the health professions, potentially pressuring an epistemic shift. Ensuring such a critical mass will require change at earlier levels, facilitating entry of Black learners into post-secondary education and providing supports for retention. It also demands thorough scrutiny of existing curricula in the health professions, to decentre Whiteness, making space for epistemic multiplicity, making space for notions that valid knowledge and valid knowers need not be White (Paton et al., 2020). It demands the movement of Black health professionals into positions of power in institutions, management positions, to embody the notion of Black knowers as credible authorities (Mpalirwa et al., 2020).

We join others in noting that the responsibility for change lies with everyone, not just Black health professionals (Salles et al., 2021). White colleagues may benefit from continuing professional development to recognize epistemic racism and understand how it operates, as well as from ‘upstander training,’ learning how to effectively disrupt and intervene (Salles et al., 2021). As Nixon (2019) has stressed in physiotherapy, critical allyship is something White colleagues must take up as a practice (not an identity), tackling the ways White supremacy is built into the everyday ways of being, thinking and knowing in the health professions. It requires learning, skillful listening, accepting guidance from racialized colleagues and building respect for their expertise into everyday interactions as well as institutional practices. Cultivating greater tolerance for discomfort among White health professionals might also be helpful, as ‘White fragility’ – an immediate hurt/anger/defensiveness when racism is raised (DiAngelo, 2011) – hinders Black colleagues who might otherwise be freer to articulate their expertise regarding racism.

Alongside peer support, mentoring, communities of practice and promotion of Black women into positions of authority, changes like this could begin to challenge epistemic racism. But they require institutional support. From educational curricula and pedagogies, to hiring and promotion, to imagery on facility walls – those of us in the health professions need to keep asking whose knowledges do we recognize and whose do we dismiss? On what bases? What counts as legitimate knowledge? What do we expect it to look like? Who is positioned as legitimate knower? How are our (‘objective, neutral’) standards for assessing knowledge claims infused with Whiteness?

## Conclusion

Epistemic racism establishes White Euro-Canadian knowers and knowledge systems as legitimate, invalidating all others. Anti-Black epistemic racism is a mechanism through which the knowledge claims, expertise, authority and professional identity of Black health professionals are undermined. This may be particularly true for Black women. As a result of this credibility deficit, Black health professionals must reconstitute their own credibility, authority, legitimacy, through impression management, exhausting extra work, or both. Meanwhile the augmented knowledges they may bring to their professions – through lived experience in marginalized communities, through lived experience of racism, through hard-won insights into how oppression operates that would benefit their clients/patients, their colleagues and their professions – is undermined or even discounted. Steps towards countering the culture of Whiteness within the health professions include embracing epistemological multiplicity, strongly supporting subordinated racialized knowledges and ways of knowing and supporting the authority of Black women knowers, especially in leadership positions.
